# An experimental assessment of algal calcification as a potential source of atmospheric CO_2_

**DOI:** 10.1371/journal.pone.0231971

**Published:** 2020-04-29

**Authors:** Olivia J. Kalokora, Amelia S. Buriyo, Maria E. Asplund, Martin Gullström, Matern S. P. Mtolera, Mats Björk

**Affiliations:** 1 Dar es Salaam University College of Education (DUCE), Dar es Salaam, Tanzania; 2 Department of Botany, University of Dar es Salaam, Dar es Salaam, Tanzania; 3 Department of Marine Sciences, University of Gothenburg, Kristineberg, Fiskebäckskil, Sweden; 4 Department of Ecology, Environment and Plant Sciences, Stockholm University, Stockholm, Sweden; 5 Department of Biological and Environmental Sciences, University of Gothenburg, Kristineberg, Fiskebäckskil, Sweden; 6 Institute of Marine Sciences, University of Dar es Salaam, Zanzibar, Tanzania; Università della Calabria, ITALY

## Abstract

Marine vegetated ecosystems such as seagrass meadows are increasingly acknowledged as important carbon sinks based on their ability to capture and store atmospheric carbon dioxide, thereby contributing to climate change mitigation. Most studies on carbon storage in marine ecosystems have focused on organic carbon, leaving inorganic carbon processes such as calcification unaccounted for, despite of their critical role in the global carbon budget. This is probably because of uncertainties regarding the role of calcification in marine carbon budgets as either atmospheric CO_2_ source or sink. Here, we conducted a laboratory experiment to investigate the influence of a calcifying alga (*Corallina officinalis* L.) on seawater carbon content, using a non-calcifying alga (*Ulva lactuca* L.) as a control. In a first part, algae were incubated separately while measuring changes in seawater pH, total alkalinity (TA) and total dissolved inorganic carbon (DIC). The amount of carbon used in photosynthetic uptake and production of CaCO_3_ was then calculated. In a second, directly following, part the algae were removed and DIC levels were allowed to equilibrate with air until the pH stabilized and the loss of CO_2_ to air was calculated as the difference in total DIC from the start of part one, to the end of the second part. The results showed that *C*. *officinalis* caused a significant and persistent reduction in total dissolved inorganic carbon (DIC), TA and seawater pH, while no such permanent changes were caused by *U*. *lactuca*. These findings indicate that calcification can release a significant amount of CO_2_ to the atmosphere and thereby possibly counteract the carbon sequestration in marine vegetated ecosystems if this CO_2_ is not re-fixed in the system. Our research emphasises the importance of considering algal calcification in future assessments on carbon storage in coastal areas.

## Introduction

Recently, there has been a considerable interest in quantifying the capacity of natural ecosystems to trap and store carbon to offset anthropogenic carbon emissions to the atmosphere [1]. Marine vegetated ecosystems such as salt marshes, mangrove forests and seagrass meadows have been identified as major carbon sinks, which store 10–18% of the total carbon stored in the ocean [2, 3]. Therefore, efforts are made to include carbon stored in the vegetated marine ecosystems in the global carbon offset schemes [4].

Earlier studies on carbon storage in vegetated marine ecosystems have mainly focused on the organic carbon only, leaving the inorganic carbon unaccounted for [1, 4]. Marine carbon sinks were assumed to consist of only photosynthesizing plants, such as seagrasses, which store excess photosynthetic products in their belowground root-rhizome system and underlying sediment [5]. However, many ecosystems, especially those in the tropics, are heavily intermixed with a wide range of calcifying organisms, such as calcareous macroalgae, and can therefore be considered hotspots for carbonate production through biological calcification [1, 6]. When marine plants remove CO_2_ from the water by photosynthesis, pH will increase correspondingly as the total inorganic carbon amount (mostly carbonate alkalinity) decreases, and the total alkalinity (TA) is thereby not affected. The total amount of carbon can then return to its initial level if the water-air equilibrium is restored and TA has remained constant. Calcifying algae, contrastingly, remove CO_2_ from water while building CaCO_3_(s), which decreases both pH and carbonate alkalinity and hence reduces TA by two equivalents for every mol of CaCO_3_ precipitated [7]. This means that when water where calcification has occurred is equilibrating with air, the levels of pH and TA will remain lower, and the total amount of carbon in the water will not return to its initial start value. Thus, CaCO_3_ precipitation induces changes in the seawater inorganic carbon system resulting in the eventual release of dissolved CO_2_ from seawater to the atmosphere [8, 9]. While the theoretical ratio of 1:1 relationship between CaCO_3_ precipitated and CO_2_ released is nearly true in freshwater, it is not so in seawater due to its buffering capacity. As a general rule, the molar ratio of CO_2_ released for every mol of CaCO_3_ precipitated is 0.6 at 25 °C and standard seawater conditions (i.e. pCO_2_, = 356 μatm, TA = 2370 pEq.kg^-1’^, and Salinity = 35) [8, 9]. Frankignoulle et al. [9] further highlighted that the level of CO_2_ released from calcification can rise with increasing seawater pCO_2_, and therefore the anticipated increase in atmospheric CO_2_ will probably lead to the release of even more CO_2_ from calcification. In many calcifying organisms, like calcareous algae, a release of CO_2_ from calcification will occur simultaneously with an uptake of CO_2_ by photosynthesis and a release of CO_2_ from mitochondrial respiration. Changes in CO_2_ from photosynthesis and respiration will, however, not affect TA, but they can affect pH and the calcium carbonate saturation state (Ω), and thus the rate of calcification [11]. This could also affect the ratio of CO_2_ lost per C fixed into CaCO_3_ (Ψ) [9]. The CO_2_ released from calcifying organisms can also be utilized in photosynthesis by nearby plants, and increase vegetated ecosystem productivity [10, 11], thereby contributing to climate change mitigation by increasing the sediment carbon storage potential [12]. Schneider and Ere [13] showed enhanced rates of photosynthesis by CO_2_ released from calcification on coral reefs, but little is known about the effect of this process on productivity and carbon storage in vegetated marine ecosystems such as seagrass meadows.

Despite the fact that calcification plays a critical role in the global carbon budget over geological time scales in calcium carbonate deposits [4], information on its role for the marine carbon budget as either source (releasing CO_2_ to the atmosphere) or sink (absorbing CO_2_ from the atmosphere) has been controversial. Brandano et al. [14, 15] reported calcification to play a key role in carbon storage, while other studies found calcification to be a source of atmospheric CO_2_, based on the stoichiometry of the chemical reactions [8, 9, 16]. The stoichiometry of the calcification reaction is complex, especially in seawater due to the strong buffering capacity, which in turn is influenced by different factors such as the total carbon concentration in the seawater [16]. This has triggered uncertainties on the role of calcification for the marine carbon cycle budget and highlights the importance of investigating the role of calcification in controlled laboratory experiments using detailed measurements of TA and DIC [4]. An advanced understanding of the role of the calcification process in algae for the marine carbon budget will improve estimations on the amount of carbon stored in the marine vegetated ecosystems. This knowledge can be used to support the evidence-based management dialogue with policy- and decision makers in view of mitigating the impact of human-induced climate change.

The present study aims to increase the understanding of the calcification process in carbon budgets in marine ecosystems. The study was designed to experimentally measure the net effect of calcification by a calcareous alga on seawater carbon content. We hypothesized that calcification of a calcareous alga would decrease both total alkalinity (TA) and pH, thereby reducing the total amount of dissolved inorganic carbon (DIC) that the seawater can hold in equilibrium with the atmosphere, eventually causing a permanent loss of inorganic carbon from the seawater surrounding the alga. We also hypothesized that the uptake of inorganic carbon by photosynthesis would increase pH, but not affect TA, and thus has no significant effects on the levels of seawater DIC after equilibrium with the atmosphere. A calcifying red macroalgae (*Corallina officinalis*) was used as a model organism in the experiment and was compared with a non-calcifying green macroalgae (*Ulva lactuca*).

## Materials and methods

### Ethics statement

No specific permits were required for the field sampling and the experiments did not involve endangered or protected species.

### Plant material and sampling

Representative specimens of two commonly occurring macroalgae, one calcifying, *Corallina officinalis* L., and another non-calcifying, *Ulva lactuca* L., were collected from the same area, at about 0 to 1 m depth in the outer part of Gullmarsfjorden on the Swedish Skagerrak coast (58°20´-58°30´N, 11°40´-11°50´E). All specimens of both species were treated in the same way and immediately transported to the laboratory, where they were cleaned from epiphytes and kept in dim light and running natural seawater to acclimatize before use in experiments. Since seawater was flowing directly from the sea into the aquaria through a flow-through system, the levels of salinity, temperature, pH and dissolved oxygen followed natural fluctuations.

### Experimental design

Laboratory experiments were conducted at Kristineberg marine research station on the Swedish west coast in August 2018. The experiments were set using 1 L incubation flasks filled with a known volume of filtered natural seawater equilibrated with air, and closed by stoppers. Each flask was placed on a magnetic stirrer to increase mixing of water and reduce the diffusion boundary layer. The experiment comprised of two algal treatments (with a known weight of algae added to each flask), i.e. one with *C*. *officinalis* and another one with *U*. *lactuca*, as well as one blank treatment consisting of only seawater, each in triplicate. The bottles were illuminated by Light Emitting Diode (LED) bars, giving a Photosynthetically Active Radiation (PAR) value of 100 μmol photons m^-2^ s^-1^ measured using a LI-Cor LI-100 DataLogger (Lincoln Nebraska, USA) with a LI-Cor Quantum Sensor. To be able to calculate the changes in DIC, and the amount of carbon utilized by calcification, measurements of pH and TA were made at different stages of the incubations. In addition, the water temperature and salinity were measured. Differences in water chemistry were estimated as the differences between the incubations with algae and the blank treatment consisting of only seawater without algae.

The incubations were made in two parts: Part 1, where the two algal species (and water only) were incubated separately in closed flasks, and Part 2, where the algae had been removed and the water was allowed to equilibrate with air in open flasks bubbled with controlled pressurised air. At the start of Part 1, seawater pH, temperature and salinity were measured in each flask using a pH electrode (Sen Tix 940, WTW) and a conductivity cell (TetraCon 925, WTW). Both electrodes also measured the temperature and were connected to a multi-meter (3430 WTW, Weilheim, Germany). Three water samples of 25 ml each were directly taken from each flask for TA measurement. The algae were then incubated to allow changes in seawater pH as the result of photosynthesis, respiration and calcification [17]. During the experiment, the position of the flasks were switched regularly to avoid any position effect and to ensure that they received equal amount of light. Measurement of seawater pH, temperature and salinity together with collecting water samples for TA measurements continued throughout the experiment at one hour interval. The incubation was stopped when the pH had risen to approximately 8.4–8.7, and thereafter in each flask final seawater pH, temperature and salinity were measured and TA samples were taken. Directly after this incubation, Part 2 was started after the algae had been filtered out and controlled pressurised air was bubbled in each flask (now without algae) to allow the seawater to equilibrate with the air until pH values were stable and had remained constant for 3–4 hours. The air used for bubbling was moisturized by passing it through a flask filled with distilled water to avoid air from drying up the seawater in the flasks. Measurements of pH, temperature and salinity continued during equilibration. As algae had been removed, no calcification or changes in TA should occur, and pH was here used as a proxy for DIC changes [18] during the equilibration with air. After stabilization, when pH remained stable, the final samples for TA measurement were taken. Collected samples were immediately filtered using 0.45μm Millipore filters and TA was measured within two days.

### Measurements of total alkalinity and DIC

TA was measured using an automatic titrator (TitroLine alpha plus, SI-analytic Mainz, Germany) with titration software (Alpha Plus version 3.1.5) from 25 ml seawater samples collected from each flask during the experiments. The equipment was made available at Kristineberg marine research station. Seawater total DIC was calculated from TA and pH using the software CO_2_ sys.xls v. 10 [19].

### Calculations of carbon uptake and loss

#### C uptake into calcification

There is a clear correlation between TA and calcification, where TA is lowered by 2 equivalents for each mole of CaCO_3_ precipitated. Thus, the total DIC content of seawater is lowered by 0.5 moles for each equivalent of TA reduction [18]. Therefore, we estimated the amount of carbon fixed into CaCO_3_ by calcification as the change in TA during the incubation with algae (Part 1) divided by 2:

$$Calcification=\frac{TA(start\;of\;Part1)-TA(end\;of\;Part1)}{2}$$ (1)

#### C uptake into photosynthesis

By assuming that calcification and photosynthetic inorganic carbon uptake was the only significant cause for carbon removal from seawater (when the full incubation flasks were closed with stoppers), the photosynthetic inorganic carbon uptake could be deducted from the changes in total inorganic carbon minus calcification:

Photosyntheticuptake=(DIC(startofPart1)-DIC(endPart1))-Calcification (2)

#### C lost to air

Since carbon removed by photosynthetic uptake (Part 1) is replenished from air during equilibration with air (Part 2), the amount of carbon driven from seawater was estimated as the total DIC at the start of Part 1, minus the final DIC at the end of Part 2, minus calcification.

Closttoair=(DIC(startofPart1)-DIC(endofPart2))-Calcification (3)

### Data analysis

Differences in pH, TA and DIC at different times of the experiment (i.e. at start, during incubation and during equilibration) as well as differences carbon uptake (or loss) were tested using one-way analysis of variance (ANOVA) in Statistica v. 13. Prior to the ANOVA analyses, the assumption of homogeneity of variances was checked by Levene’s test, and when necessary, the data was log10(x+1)-transformed or (in most cases) analysed using the non-parametric Kruskal-Wallis test was used. A posteriori multiple comparison tests were carried out with either Tukey’s test (on parametrically analysed data) or the non-parametric Mann-Whitney test. Simple linear regression analysis was used to explore the relationship between carbon uptake in photosynthesis (dependent variable) and carbon uptake in calcification (regressor) as well as to examine the relationship between the level of carbon lost (dependent variable) and carbon uptake in calcification (regressor). The statistical significance level was set at p<0.05 in all statistical analyses.

## Results

### Changes in seawater pH, total alkalinity (TA) and total dissolved inorganic carbon (DIC)

**Fig 1:**
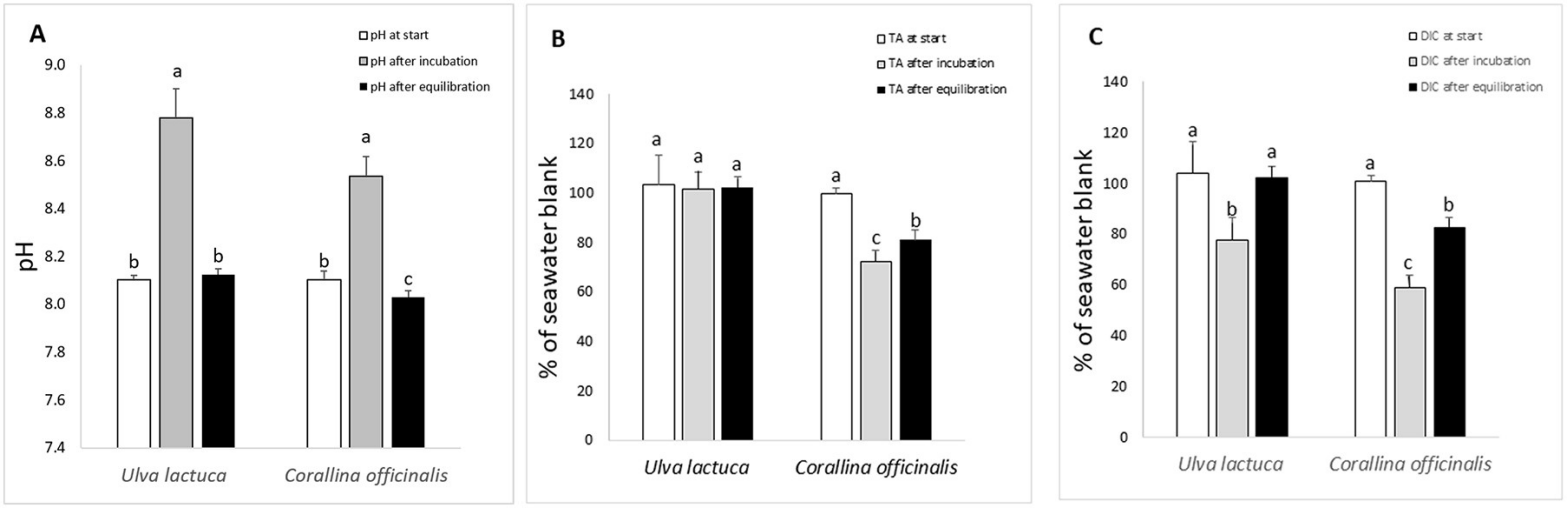
Changes in seawater pH (A), total alkalinity (B), and total inorganic carbon (C) in *U*. *lactuca* and *C*. *officinalis* incubations, at different times of the experiment. Letters a, b and c (above error bars) indicate significant differences (p<0.05) between treatments. Values are averages ±SD, n = 17.

Changes in seawater pH, TA and DIC during the experiment are illustrated in Fig 1. In both treatments, the pH increased during incubation. After equilibration with air, the pH dropped in both treatments and returned to its start value in the *U*. *lactuca* treatment, while in the *C*. *officinalis* treatment the end pH was significantly lower than the start value (Mann-Whitney U test, p<0.001, Fig 1a). No changes in TA were found in *U*. *lactuca* (Fig 1b), but in *C*. *officinalis* the TA decreased significantly during incubation (Tukey’s test, p<0.001, Fig 1b). During equilibration, the TA in the *C*. *officinalis* treatment increased slightly, but remained significantly lower than the start value (Tukey’s test, p<0.001, Fig 1b). Similarly, during incubation, the DIC in both treatments decreased significantly during incubation (Mann-Whitney U test, p<0.001, Fig 1c). During equilibration, DIC increased and returned to its start value in *U*. *lactuca*, but in *C*. *officinalis* DIC remained significantly lower than the start value (Mann-Whitney U test, p<0.001, Fig 1c).

### Carbon removal from the water by photosynthesis and calcification

**Fig 2:**
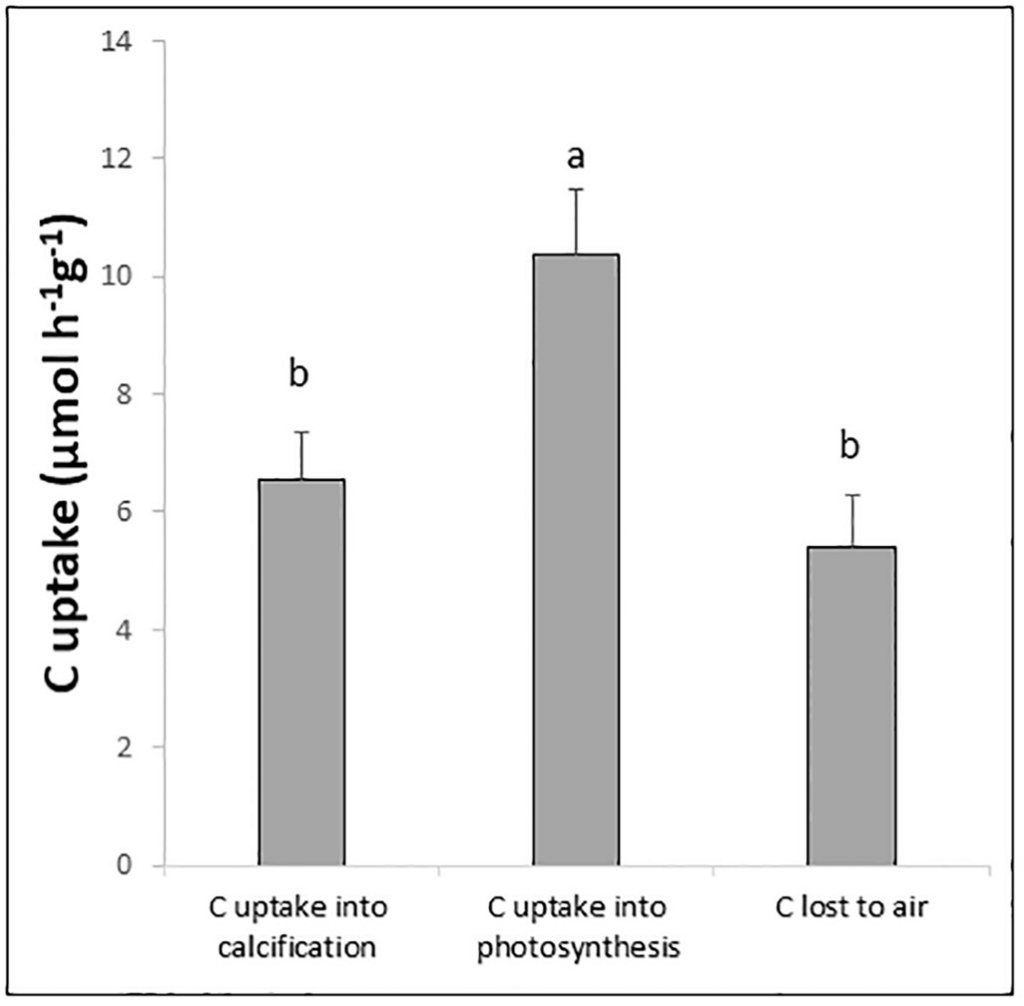
Carbon removal from seawater by calcification, photosynthesis and carbon lost to the air in the *C*. *officinalis* treatment during incubation. Letters (below error bars) indicate significant differences (p<0.05) between treatments. Values are averages ± SD, n = 17.

Carbon was removed from the water by *C*. *officinalis* through both photosynthesis and calcification as well as via carbon loss to the air (Fig 2). For *U*. *lactuca*, removal of carbon was only through photosynthetic uptake, and the rate of uptake was higher for *U*. *lactuca* than for *C*. *officinalis* (39.1 ± 2.6 and 10.4 ± 1.1, respectively). Photosynthetic carbon uptake was significantly higher than both carbon uptake into calcification (ANOVA; p<0.05) and carbon lost to the air (ANOVA; p<0.01) in *C*. *officinalis*. The level of carbon removal into calcification and the level of carbon lost to the air did not significantly differ from each other.

### Estimation of carbon loss from seawater by calcification

**Fig 3:**
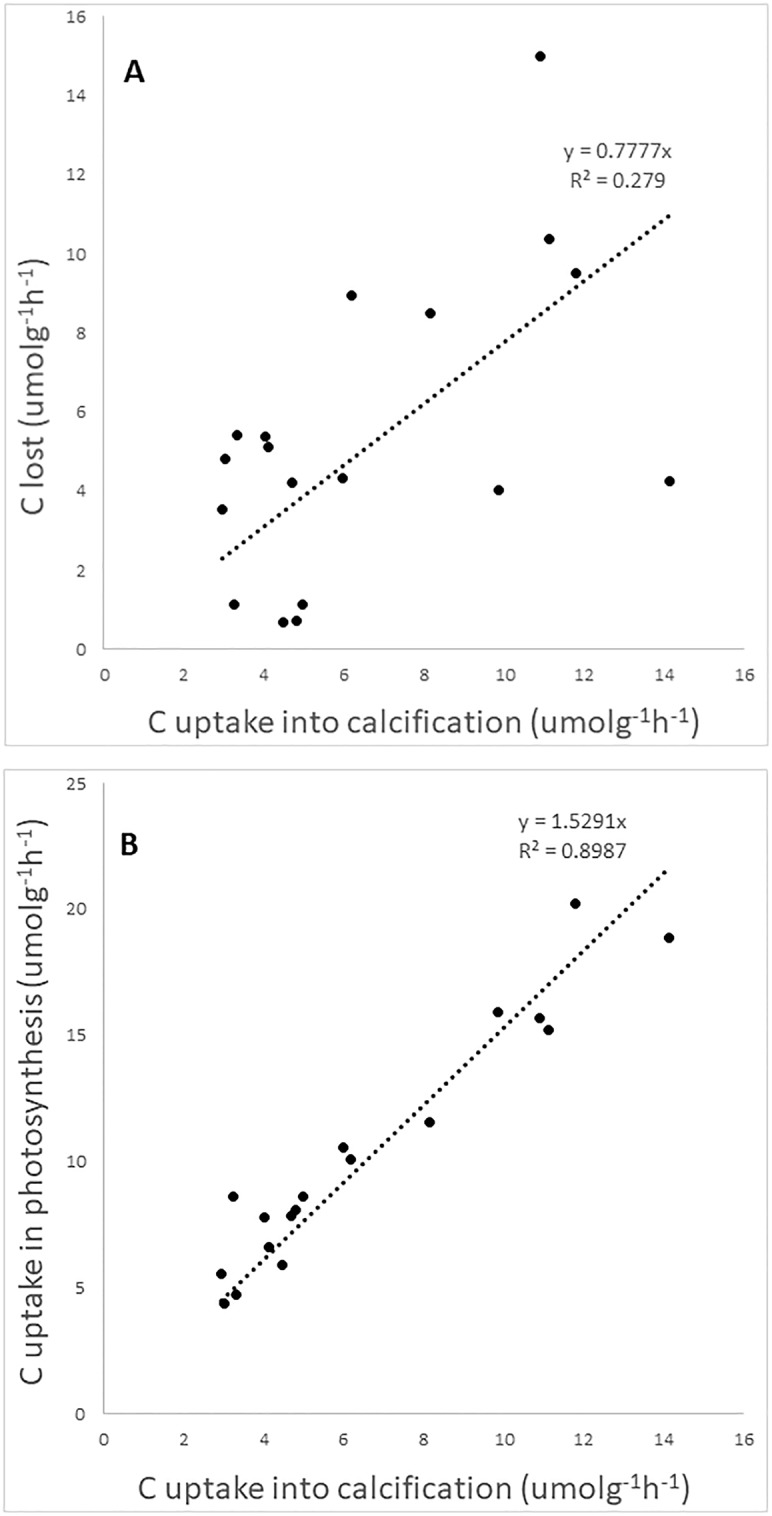
The correlation between carbon uptake via calcification and carbon lost to air by *C*. *officinalis* during incubation (A) and between carbon fixed by calcification and carbon removal by photosynthesis (B). The dotted lines indicate linear regressions. All values shown.

The proportion of carbon fixed by calcification was positively related to the amount of CO_2_ lost to the air (Linear regression, p < 0.05; Fig 3a). The estimated ratio-value was 0.78, close to the theoretical ratio-value of 0.6 reported in previous studies [8, 9, 16] even though variation was high. There was a clear positive correlation between the amount of carbon fixed in photosynthesis and the amount fixed in calcification (Linear regression, p < 0.001; Fig 3b), with a ratio of approximately 1:1.5, so that for every mol CO_2_ fixed in calcification, 1.5 mol was fixed in photosynthesis.

## Discussion

This study showed that calcification of a calcareous red algae caused a net reduction of seawater total alkalinity (TA) and pH, eventually driving CO_2_ from the seawater to the atmosphere. The photosynthetic removal of CO_2_ did, however, not affect the amount of inorganic carbon in the seawater after it has been allowed to equilibrate with air.

Both algae were capable of raising the seawater pH during incubation due to a net photosynthetic uptake of inorganic carbon, as shown by the decreases in total dissolved inorganic carbon (DIC). As revealed by the differences in DIC and TA between the two algae, inorganic carbon uptake in *U*. *lactuca* was caused by photosynthetic removal only (since DIC was lowered during incubation but not TA), whereas in *C*. *officinalis* the uptake was caused by both net photosynthetic carbon fixation and calcification (as both DIC and TA were lowered during the incubation phase). The subsequent equilibration with air caused a drop in seawater pH as water DIC was replenished from CO_2_ in the air. The pH of the water returned to its start value in *U*. *lactuca*, but remained significantly lower in the water surrounding *C*. *officinalis*, where also TA and DIC levels were permanently lowered. This shows the capacity of calcareous algae to drive CO_2_ from the seawater to the atmosphere [8, 9] and agrees with *in situ* studies on coral reefs showing that areas dominated by calcifying corals elevate pCO_2_, whereas non-calcifying algal beds draw CO_2_ from the atmosphere (potentially offsetting ocean acidification impacts at the reef scale) [17, 20]. In our experiments, about 0.78 mol of CO_2_ was found to be lost to air for every mol CO_2_ fixed in calcification. This ratio is not dramatically different from the calculated 0.6 suggested previously [8, 9, 16]. We should, however, stress that even though all incubations with *C*. *officinalis* resulted in an eventual loss of CO_2_ to the atmosphere, the amounts varied substantially and we are not able to define an exact ratio of carbon lost per carbon used into calcification. There was a strong positive correlation (R^2^ = 0.9) between calcification and photosynthetic carbon uptake in *C*. *officinalis*, indicating the commonly suggested link between the two processes (see e.g. [21]). Our results show that 1.5 mol carbon was taken up by photosynthesis for every mol carbon fixed into calcification. Previous studies have reported similar rates, e.g. a ratio of approximately 1:1 for *C*. *officinalis* [22], and a ratio of around 1.6 mol C taken up by photosynthesis for every mol C fixed into calcification for four deep-water *Halimeda* species [23].

Even if CO_2_ is released from calcifying algae into the seawater, it does not necessarily have to reach the atmosphere if other efficient photosynthetic organisms are present in the vicinity. In tropical areas, calcareous macroalgae often thrive within dense seagrass meadows [6, 24], forming productive seagrass-dominated meadows, which in turn provide important ecological goods and services [25, 26]. In shallow seagrass meadows, inorganic carbon consumption by seagrass photosynthesis has been reported to increase seawater pH drastically, which enhances macroalgal CaCO_3_ precipitation [11]. On the other hand, high seawater pH reduces seagrass photosynthesis [27], because at high pH, most of the dissolved inorganic carbon is forced into forms inaccessible to plants [28, 29]. If CO_2_ released from calcification is not escaping to the atmosphere, it might be fixed by surrounding plants and associated epiphytes during photosynthesis [10, 30]. Therefore, the released CO_2_ might play a key role of increasing community productivity, most likely at high pH and especially in carbon-limited environments such as tropical seagrass meadows [10, 31], thus, leading to increased carbon sequestration in these areas [12]. To what extent such positive feedback from calcification to productivity occurs in situ is, however, not known, and it has to be considered that this increased productivity will keep pH at high levels and thus also keep up calcification [32].

Our findings show that algal calcification can cause loss of a significant amount of inorganic carbon from the seawater to the air and might therefore act as an atmospheric CO_2_ source. This loss of inorganic carbon may partly counteract the role of marine vegetated ecosystems, such as seagrass beds and macroalgal belts, as major carbon sinks. However, the amount of CO_2_ from calcification that actually escapes to the atmosphere or is being recycled within the system by photosynthetic organisms will be highly controlled by different local environmental factors and the level of surrounding ecosystem productivity. Therefore, further studies on carbon storage in vegetated marine ecosystems that account for calcification are crucial to improved estimates of carbon storage for protection and management purposes to mitigate human-induced climate change.

## Supporting information

S1 Data (XLSX)
